# Postoperative Outcomes and Risk Profiles in Dual Liver–Lung Transplantation: A Single‐Center Retrospective Analysis

**DOI:** 10.1155/joot/9414873

**Published:** 2026-04-08

**Authors:** Abdulmalik Saleem, Omar Ilyas, Mark Obri, Ahmad Alomari, Muhammad Saad Faisal, Haya Omeish, Ammad Chaudhary, Yara Dababneh, Uthman Shukairy, Shunji Nagai, Domingo Franco-Palacios, Deepak Venkat, Adarsh Varma, Syed-Mohammed Jafri

**Affiliations:** ^1^ Department of Internal Medicine, Henry Ford Hospital, Detroit, Michigan, USA, henryford.com; ^2^ Department of Internal Medicine, Michigan State University College of Human Medicine, East Lansing, Michigan, USA, msu.edu; ^3^ Department of Gastroenterology and Hepatology, Henry Ford Hospital, Detroit, Michigan, USA, henryford.com; ^4^ Department of Internal Medicine, Cornell University School of Medicine, Ithaca, New York, USA; ^5^ Department of Transplant Surgery, Henry Ford Hospital, Detroit, Michigan, USA, henryford.com; ^6^ Department of Pulmonary Transplant, Henry Ford Hospital, Detroit, Michigan, USA, henryford.com

**Keywords:** acute cellular rejection, biliary complications, dual liver–lung transplantation, fungal infection, immunosuppression, survival

## Abstract

**Background:**

Dual liver–lung transplantation (DLLT) is an uncommon but definitive therapy for carefully selected patients with concurrent end‐stage hepatic and pulmonary disease. The combined operative complexity and dual‐organ immunosuppressive burden may predispose recipients to early morbidity and graft‐threatening complications [1–4].

**Objective:**

To characterize early and late postoperative events (including acute cellular rejection (ACR), infectious complications, malignancy, and hospital readmissions) after DLLT and to describe associated clinical patterns.

**Methods:**

We performed a retrospective cohort study of adult DLLT recipients at a single tertiary center (2013–2024). Variables included demographics, transplant indications, ischemia times, readmissions (0–3 months; 3–12 months), infections (timing/etiology/site), biopsy‐proven rejection, malignancy, and survival. ACR was biopsy‐confirmed in cases of unexplained transaminitis beyond 30 days posttransplant. Analyses were descriptive, consistent with STROBE recommendations for small cohorts.

**Results:**

Ten patients (mean age 53.7 years; 50% female) underwent DLLT. Liver etiologies included alcohol‐related cirrhosis (*n* = 2), HCV (*n* = 1), cryptogenic (*n* = 1), autoimmune (*n* = 1), cystic fibrosis (*n* = 1), and unspecified (*n* = 4). Lung indications were IPF (*n* = 5), pulmonary hypertension (*n* = 2), ILD (*n* = 2), and CF (*n* = 1). All patients were readmitted within 90 days, most commonly for infection (40%), diarrhea (20%), critical illness myopathy (20%), rejection (10%), and biliary stricture (10%). Biopsy‐proven ACR occurred in 4/10 patients (40%) after the first month, uniformly presenting with hepatocellular transaminemia; 3/4 received pulse‐dose IV corticosteroids and 2/3 subsequently developed invasive fungal disease (Aspergillus and *Candida*). Overall, 9/10 experienced infection within 6 months, predominantly pulmonary (fungal/bacterial pneumonias). Three patients developed malignancy (basal cell carcinoma, prostate carcinoma, and angiosarcoma [fatal]). Survival was 90% at 1 year, 70% at 3 years, and 60% at 5 years; no 10 year survivors were observed.

**Conclusions:**

DLLT is associated with early readmission and a high infectious burden, particularly invasive fungal disease after steroid‐treated ACR. Despite significant early morbidity, short‐term survival is favorable. Multicenter studies are needed to refine candidate selection, balance rejection prophylaxis with antifungal strategies, and standardize long‐term oncologic and dermatologic surveillance in DLLT.

## 1. Introduction

Dual liver–lung transplantation (DLLT) is an innovative and therapeutic pathway for patients with advanced disease in both organs and a need for immediate attention to both. This includes patients with cystic fibrosis (CF), idiopathic pulmonary fibrosis (IPF) with cirrhosis, and pulmonary hypertension with decompensated liver disease [1–4]. From a hepatology standpoint, DLLT imposes a distinctive risk profile, where the liver graft is vulnerable to an ischemia‐reperfusion injury, early cholestatic or hepatocellular allograft dysfunction, and biliary complications [5]. From a pulmonology standpoint, the lung graft has a high susceptibility to infection and allograft dysfunction [6]. This interplay exemplifies the complex immunosuppressive management strategy required to balance lung rejection risk against hepatic toxicity and infectious complications. Overt immunosuppression for lung graft management can create an increased risk for hepatic toxicity [2–4]. A liver‐first operative strategy is often favored in CF and certain portal hypertensive states, though optimal sequencing and perioperative immunosuppression are incompletely defined [2].

Despite technical feasibility, DLLT remains one of the most complex procedures in solid organ transplantation, requiring prolonged operative times, coordination between thoracic and abdominal surgical teams, and careful hemodynamic management. With few centers performing DLLT, the literature remains limited to small series, registry reviews, and isolated case reports, leaving major gaps in understanding of readmission burden, rejection phenotypes, biliary outcomes, and malignancy risk in this highly selected population.

This study seeks to address these gaps by reporting the outcomes of DLLT recipients at a single large tertiary transplant center over an eleven‐year period. We specifically characterize early and late readmissions, acute cellular rejection (ACR) patterns, infection profiles, and malignancy development, while reporting overall survival. By highlighting hepatology‐relevant complications such as transaminemia, biliary strictures, and infection‐rejection tradeoffs, we aim to provide granular data that can inform patient selection, immunosuppressive tailoring, and long‐term surveillance strategies in DLLT.

## 2. Methods

### 2.1. Study Design and Population

We conducted a retrospective cohort study of all adult DLLT recipients at Henry Ford Health from January 2013 to December 2024. Inclusion criteria included patients aged ≥ 18 years and receipt of combined liver and lung allografts. Exclusion criteria for our study included patients who did not undergo DLLT. The study is reported in accordance with STROBE guidelines for observational studies.

### 2.2. Variables and Definitions

Data were collected from the electronic health record. Baseline demographic variables including age, sex, race/ethnicity, and indications for liver and lung transplantation were collected. Data regarding outcomes including hospital readmissions (0–3 months, 3–12 months, and  > 12 months), infections (timing/etiology/site), incidence of ACR, incidence of malignancy, and mortality (1, 3, 5, and 10 years) were collected. Rejection was defined by biopsy‐proven pathology. Subsequent management was also recorded. Infection was recorded postoperatively at 0–1 months and 1–6 months posttransplant. Infections after 6 months were unable to be recorded due to variable follow‐up.

### 2.3. Statistical Approach

Given the sample size, analyses were descriptive (counts, proportions, and time‐anchored summaries). No multivariate modeling was performed to avoid false inference.

## 3. Results

### 3.1. Cohort Characteristics

Ten recipients (5 male, 5 female; mean age 53.7 years) underwent DLLT. Ten out of ten patients were White. Liver disease etiologies were alcohol‐related (20%), HCV (10%), cryptogenic (10%), autoimmune (10%), CF (10%), and unspecified (40%). Lung indications were IPF (50%), pulmonary hypertension (20%), ILD (20%), and CF (10%).

### 3.2. Readmissions and Early Morbidity

All patients were readmitted within 90 days. Leading etiologies were infection (4/10), diarrhea (2/10), critical illness myopathy (2/10), biopsy‐proven rejection (1/10), and biliary stricture (1/10). Between 3 and 12 months, atrial fibrillation with RVR, hypoxia, liver rejection, and diarrhea were the causes of the 4 patients who were admitted. Those who were readmitted in the 1–5‐year period was due to atrial fibrillation with RVR, liver transplant complication, seroma/abscess, abnormal MRCP, pancytopenia, and acute respiratory distress due to an outpatient ERCP.

### 3.3. Rejection

ACR occurred in 4/10 (40%) after the first month of transplant. All four patients presented with elevated liver function tests. All patients received IV Solu‐Medrol for three days with two patients receiving an oral prednisone taper. Normalization of liver enzymes was achieved in all patients after steroid course with no episodes of recurrence. No episodes of lung graft rejection were noted in our cohort. Mean time after transplant for rejection was 89 days after transplant.

### 3.4. Infectious Complications

Three out of ten patients in our cohort had culture or biopsy‐proven infection within a month of transplant. All three infections were bacterial in nature including Staphylococcus aureus pneumonia, Aspergillus pneumonia, and pseudomonas pneumonia. Six patients developed infections between one and six months posttransplant, 2 patients developed pseudomonas pneumonia, one patient developed a chest wall abscess with MSSA and sternal osteomyelitis, another *E. faecium* empyema with *Candida seroma*, one CMV viremia, and one *Candida glabrata* empyema.

### 3.5. Malignancy

Three patients developed de novo malignancy post DLLT: basal cell carcinoma at 3 years (treated with Mohs), prostate carcinoma at 2 years (robotic prostatectomy), and angiosarcoma at 9 years (hospitalization leading to death from sepsis). These events highlight the oncologic signal under chronic calcineurin‐based immunosuppression and intermittent high‐dose corticosteroids.

### 3.6. Survival

Observed survival was 9/10 at 1 year, 2/7 at 3 years, 3/5 at 5 years, and 0/1 at 10 years. To note, the first recorded transplant took place in 2013 and was the only transplant to have been performed 10 years prior to data collection. Two out of three causes of mortality were multifocal pneumonia resulting acute hypoxic respiratory failure and one was septic shock secondary to presumed fungemia. The average time of mortality after transplant was 1,774.67 days.

## 4. Discussion

Our single‐center experience of DLLT demonstrated universal early readmissions, high rates of infectious complications, and a notable burden of ACR, yet overall survival remained acceptable. These findings demonstrate both the feasibility of DLLT and the ongoing need to refine perioperative and long‐term management strategies in this high‐risk population.

Compared to prior reports, the infectious burden in our cohort was strikingly high, with 70% of patients experiencing infection within six months. This exceeds infection rates reported in isolated liver transplantation, which typically range from 45% to 56% within the first year, where bacterial infections are more common, and fungal complications are less frequent [7]. Our data are consistent with previous reports in liver transplantation where bacterial pathogens predominate in the first month of transplant [8]. Of the 3 patients found to be infected in the first month, two had bacterial pneumonia, while the third had fungal pneumonia. This is similar to the results found in the existing literature [8]. In our cohort, we continued to see bacterial infections predominating through the 1–6 month period as well. Of the 6 infected, 4 were found to have pneumonia with bacterial etiology. In line with established research, our cohort also saw opportunistic infections such as CMV viremia and multiple fungal infections within the same 1–6‐month period [7]. There are no specific reports in the literature for infections and infectious admission one year post combined dual lung–liver transplant; however, our findings highlight that infection is a leading cause of comorbidity in this population similar to what is reported in isolated liver transplantations [9].

The higher reported infection rate one year postoperatively may be due to a few factors. Patients undergoing DLLT may be on more aggressive immunosuppressive regimens, increasing susceptibility to nosocomial and opportunistic infections [10, 11]. Furthermore, the presence of two allografts may increase the chances of donor‐derived infections through a potential increase in a nidus for infection [12].

In contrast, the predominance of fungal pneumonia and empyema in our DLLT cohort parallels the high infectious susceptibility described in lung‐alone transplantation, where invasive fungal disease remains a leading cause of early morbidity and mortality [13]. Notably, two of our patients developed fungal infections shortly after pulse corticosteroid therapy for rejection, illustrating the infection–rejection tradeoff that is particularly pronounced in DLLT.

ACR occurred in 10% of recipients in the first month and in 40% of recipients after the first month posttransplant, manifesting exclusively with hepatocellular transaminemia with no lung rejection recorded in our cohort (Table 1). This rate appears higher than typically reported in isolated liver transplantation, where early rejection within one year has been reported between 10% and 35% [14, 15]. Our rate of rejection was also elevated compared to isolated lung transplantation, which was reported between 20% and 50% [16–18].

**TABLE 1:** Rejection events by posttransplant interval.

Patient	Rejection 0–1 month	Rejection 1–6 months
1	No	No
2	No	No
3	No	ACR
4	No	No
5	No	No
6	No	ACR
7	No	ACR
8	ACR	No
9	No	No
10	No	ACR

Abbreviation: ACR, acute cellular rejection.

The existing literature indicates that DLLT patients are reported to have lower rates of rejection, with one cohort study reporting two episodes of rejection both noted in the lung [11]. In a single center and registry analysis, freedom from biopsy‐proven lung rejection at the five‐year mark was reported at 91% compared to 50% isolated liver rejection [19]. Interestingly, only one other report has demonstrated biopsy‐proven liver rejection after DLLT, as all other forms of rejection in this population have been reported in the lung [20].

The liver’s immunoprotective effects stem from its rich population of donor‐derived antigen presenting cells as well as its capacity to induce regulatory T‐cells and promote immune deviation, anergy, and apoptosis of alloreactive T‐cells. The organ’s architecture confers abortive activation and exhaustion of recipient T‐cells yielding immune tolerance [21–25]. This is evident in the literature as liver transplantation with another organ, such as the heart or the kidney, confers lower rates of ACR in the nonhepatic allograft compared to isolated transplantation. For example, in heart–liver transplantation, there is a markedly reduced rate of cardiac allograft vasculopathy, and T‐cell‐mediated rejection [26].

The difference in our cohort can potentially be attributed secondary to perioperative inflammation or ischemia perfusion injury which may disrupt the liver’s tolerogenic environment [27, 28]. Furthermore, particularly in the case of liver–lung transplantation, the lung allograft is particularly immunogenic and necessitates immunosuppression, paradoxically predisposing to under or over immunosuppression potentially increasing the risk for rejection in the liver allograft [10, 11, 29].

Importantly, while rejection resolved biochemically in all patients treated with intravenous steroids, the subsequent development of invasive fungal disease in half of these cases reflects a unique DLLT‐specific vulnerability. Unlike lung‐alone transplantation, where rejection surveillance is guided by transbronchial biopsy and treatment protocols are standardized, rejection in DLLT presents heterogeneously and is complicated by overlapping hepatic and pulmonary immunologic triggers. These findings highlight the need for center‐specific immunosuppression strategies that minimize steroid exposure without compromising graft survival.

The causes of mortality in our series were primarily infectious; however, they occurred in the window that patients are typically more prone to community‐acquired infections with the average time to mortality after liver transplant being 1774 days.

The causes of mortality in our population emulate patterns established in earlier research studies, where infection and malignancy predominate over other causes. We saw 30% mortality from both multifocal pneumonias and from the development of angiosarcoma in one patient. Recent literature has demonstrated that mortality rates post‐DLLT are comparable to isolated lung transplantation, with 1‐year survival of 89.5% and 5‐year survival of 67% in matched cohorts [30]. Single‐center series and registry studies report similar findings, with 1‐year survival rates of 71.4% to 79% and 5‐year survival rates of 49% to 63% [10, 31].

We also noticed that our patients had a 100% readmission rate among the patients within the first month, with infections, diarrhea, and critical illness myopathy complications serving as leading causes within the first 1–3‐month period (Table 2). After the 3‐month period, readmissions dropped among our patient population, and the leading etiology shifted largely to infection. The diversity of readmissions mirrors prior observations in DLLT studies that indicate that early readmissions are common, but their etiologies are not clearly delineated.

**TABLE 2:** Readmissions by time interval after dual liver–lung transplantation.

*N*	0–3 months readmission	Cause (0–3 months)	3–12‐month readmission	Cause (3–12 months)	1–5‐year readmission	Cause (1–5 years)
1	Yes	Critical illness myopathy	Yes	Afib with RVR	Yes	Afib with RVR
2	Yes	Critical illness myopathy	Yes	Hypoxia	No	—
3	Yes	Chills, nausea, NBNB emesis, fatigue	Yes	Liver rejection	Yes	Liver transplant complication
4	Yes	Neutropenia, bandemia, anemia, pleural effusions, empyema	No	—	Yes	Seroma, abscess
5	Yes	Biliary anastomotic stricture	No	—	Yes	Abnormal MRCP
6	Yes	Acute hypoxic respiratory failure	No	—	No	—
7	Yes	Liver biopsy showing acute cellular rejection	No	—	Yes	Pancytopenia
8	Yes	Diarrhea 2/2 bactrim overdose	No	—	Yes	Acute respiratory distress 2/2 outpatient ERCP
9	Yes	Diarrhea	Yes	Diarrhea	No	—
10	Yes	Bilateral pleural effusions; spirometry ↓17%	No	—	No	—

*Note:* Afib = atrial fibrillation; MRCP = magnetic resonance cholangiopancreatography; ERCP = endoscopic retrograde cholangiopancreatography; NBNB = nonbilious, nonbloody; 2/2 = secondary to.

Based on our institutional experience, several lessons have been learned that may help other teams improve results after DLLT. In our cohort, universal early readmission was observed, indicating that outcomes in DLLT are strongly influenced by the early postdischarge period and may benefit from structured, multidisciplinary follow‐up. In addition, ACR consistently involved the liver graft and presented with hepatocellular transaminemia, without documented lung rejection, suggesting that timely liver‐focused evaluation and biopsy for unexplained enzyme abnormalities is critical in DLLT recipients. Infectious complications were frequent within the first six months captured in this study, and all rejection episodes were treated with high‐dose corticosteroids; the occurrence of severe infections and infection‐related mortality in this setting underscores the importance of careful immunosuppression management and heightened infectious vigilance during periods of intensified therapy. Finally, the development of de novo malignancies during follow‐up highlights the need for ongoing long‐term surveillance as part of routine DLLT care. Collectively, these observations suggest that improvements in DLLT outcomes may depend less on operative factors and more on refinements in posttransplant monitoring, immunosuppression coordination, and longitudinal multidisciplinary care, which may offer practical guidance to other centers performing this rare procedure.

This study has several notable strengths. DLLT remains exceedingly rare, and reporting a decade of institutional experience contributes meaningful data in an otherwise sparse literature. This study also has the advantage of uniquely including causes of readmission, infectious microbiology, and biopsy‐proven rejection. Furthermore, the study incorporates survival estimates through five years, allowing comparison to registry and single‐organ transplant outcomes.

There are several limitations of this study as well. The small sample size (*n* = 10) limits the statistical power and precludes multivariate analysis, making observed trends in our study difficult to generalize. The retrospective, single‐center design introduces potential bias from local practice patterns, and the heterogeneity of indications (CF, IPF, PHTN, and diverse liver diseases) complicates interpretations. Without a comparison group of isolated lung or liver transplant recipients, it is difficult to contextualize whether morbidity and mortality rates are specific to DLLT or reflect general transplant risk.

Despite these limitations, this study provides rare and detailed insights into DLLT outcomes and complications, highlighting the challenges of immunosuppression, infection risk, and long‐term surveillance. These findings underscore the necessity for multicenter collaborations to refine patient selection, optimize prophylactic strategies, and improve long‐term care for this high‐risk transplant population. DLLT is associated with high rates of early readmission, acute rejection, and particularly fungal infections, yet short‐term survival is achievable in carefully selected patients. Our findings underscore the importance of balancing rejection prophylaxis against infectious risk, particularly with high‐dose corticosteroids, and reinforce the need for long‐term dermatologic and oncologic surveillance. Multicenter studies with larger cohorts will be essential to refine candidate selection, standardize perioperative immunosuppression, and optimize long‐term outcomes in this vulnerable population (see Tables 3 and 4).

**TABLE 3:** Posttransplant infections after dual liver–lung transplantation.

Patient	Infection 0–1 months (cause)	Infection 1–6 months (cause)	Infection after DLLT	Type
1	—	Gram‐negative bacilli PNA	Yes	Bacterial
2	Staph aureus PNA	Chest wall abscess MSSA; sternal osteomyelitis	Yes	Bacterial
3	Aspergillus PNA; pleural effusion	—	Yes	Fungal
4	—	*E. faecium* empyema; *Candida*; chest wall abscess	Yes	Bacterial/fungal
5	—	—	Yes	Viral
6	Gram‐negative bacilli PNA	ESBL/PSA PNA	Yes	Bacterial
7	—	—	No	n/a
8	—	—	No	n/a
9	—	CMV viremia	Yes	Viral/bacterial
10	—	*Candida gabralta* empyema	Yes	Fungal

*Note:* HCV, Hepatitis C virus; PNA, pneumonia.

Abbreviations: ACR, acute cellular rejection; CF, cystic fibrosis; DLLT, dual liver–lung transplantation; ICU, intensive care unit; ILD, interstitial lung disease; IPF, idiopathic pulmonary fibrosis.

**TABLE 4:** Patient demographics and transplant indications.

Patient number	Age (years)	Sex	Race	Lung diagnosis	Liver diagnosis
1	60	Male	White	IPF	NA
2	60	Female	White	IPF	NA
3	55	Female	White	Pulmonary HTN	Autoimmune
4	63	Female	White	IPF	ETOH
5	66	Male	White	ILD	Cryptogenic
6	43	Female	White	ILD	NA
7	36	Male	White	CF	CF
8	68	Female	White	IPF	HCV
9	56	Male	White	Pulmonary HTN	NA
10	66	Female	White	IPF	ETOH

*Note:* HTN = hypertension, HCV = Hepatitis C virus, ETOH = alcohol, and NA: information not available.

Abbreviations: CF = cystic fibrosis, ILD = interstitial lung disease, and IPF = idiopathic pulmonary fibrosis.

## Author Contributions

All authors contributed to study design, data acquisition or interpretation, and critical manuscript revision.

## Funding

No external funding was received.

## Disclosure

All authors approved the final version.

## Ethics Statement

This study was approved by the institutional review board with waiver of informed consent for retrospective minimal‐risk research.

## Conflicts of Interest

The authors declare no conflicts of interest.

## Data Availability

Data sharing is not applicable to this article as no datasets were generated or analyzed during the current study.
